# Supplementary material for: Leukotriene B4 Receptor 2 Mediates the Production of G-CSF That Plays a Critical Role in Steroid-Resistant Neutrophilic Airway Inflammation
Source: Biomedicines. 2022 Nov 19;10(11):2979. doi: 10.3390/biomedicines10112979 (PMC9687517; doi:10.3390/biomedicines10112979)
Supplement: Supplementary file 1 [file biomedicines-10-02979-s001.zip › biomedicines-2033647-supplementary.pdf]

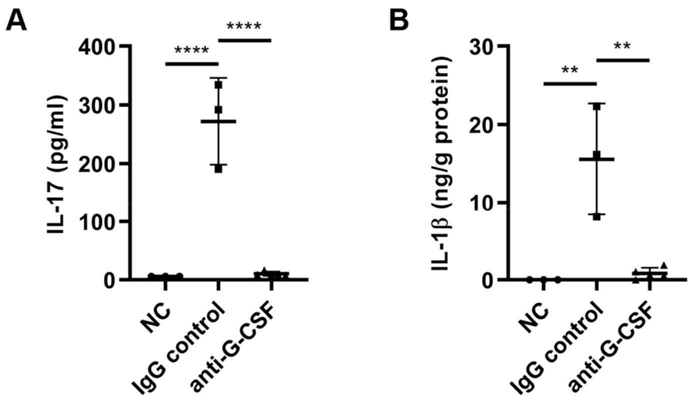

Supplementary Figure S1. G-CSF is critical for IL-17/IL-1β production in steroid-resistant neutrophilic airway inflammation. To analyze the effect of anti-G-CSF, mice were administered anti-G-CSF (5 mg/kg) or control IgG1 (5 mg/kg) by i.p. injection 1 h before each challenge (n = 3 – 5 per group). The negative controls (NC) were untreated. (**A**,**B**) IL-17 level in BALF and IL-1β level in lung lysate were analyzed by ELISA. The experiments were performed in triplicate. ** $p < 0.01$, **** $p < 0.0001$ versus each control group.

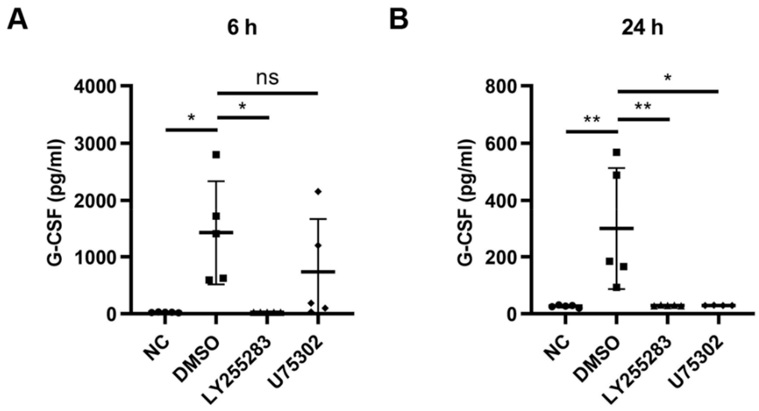

Supplementary Figure S2. BLT1 has no effect on G-CSF production in the early time point following the challenge. BLT2 antagonist LY255283 (10 mg/kg) or BLT1 antagonist U75302 (0.5 mg/kg) was administered by i.p. injection 1 h before each challenge. The negative controls (NC) were untreated. (**A**,**B**) G-CSF level in BALF was analyzed by ELISA. The experiments were performed in triplicate. * $p < 0.05$, ** $p < 0.01$ versus each control group.
